# The effect of morphology on spelling and reading accuracy: a study on Italian children

**DOI:** 10.3389/fpsyg.2014.01373

**Published:** 2014-11-19

**Authors:** Paola Angelelli, Chiara Valeria Marinelli, Cristina Burani

**Affiliations:** ^1^Department of History, Society and Human Studies, University of SalentoLecce, Italy; ^2^Istituto di Ricovero e Cura a Carattere Scientifico (IRCCS) Santa LuciaRome, Italy; ^3^Istituto di Scienze e Tecnologie della Cognizione, Consiglio Nazionale delle RicercheRome, Italy; ^4^Department of Life Sciences, University of TriesteTrieste, Italy

**Keywords:** morphology, spelling, orthography, typically developing readers/spellers, transparent orthographies

## Abstract

In opaque orthographies knowledge of morphological information helps in achieving reading and spelling accuracy. In transparent orthographies with regular print-to-sound correspondences, such as Italian, the mappings of orthography onto phonology and phonology onto orthography are in principle sufficient to read and spell most words. The present study aimed to investigate the role of morphology in the reading and spelling accuracy of Italian children as a function of school experience to determine whether morphological facilitation was present in children learning a transparent orthography. The reading and spelling performances of 15 third-grade and 15 fifth-grade typically developing children were analyzed. Children read aloud and spelled both low-frequency words and pseudowords. Low-frequency words were manipulated for the presence of morphological structure (morphemic words vs. non-derived words). Morphemic words could also vary for the frequency (high vs. low) of roots and suffixes. Pseudo-words were made up of either a real root and a real derivational suffix in a combination that does not exist in the Italian language or had no morphological constituents. Results showed that, in Italian, morphological information is a useful resource for both reading and spelling. Typically developing children benefitted from the presence of morphological structure when they read and spelled pseudowords; however, in processing low-frequency words, morphology facilitated reading but not spelling. These findings are discussed in terms of morpho-lexical access and successful cooperation between lexical and sublexical processes in reading and spelling.

## Introduction

Analysis of the corpora and databases of several languages revealed that about 60% of less familiar words are either derived by affixation or compounds (see, e.g., Nagy and Anderson, 1984; Thornton et al., 1997, for American English and Italian, respectively). Thus, a large proportion of the unfamiliar words read and spelled by children in primary school are likely to be morphologically complex (Nagy and Anderson, 1984). In the last few decades it has been frequently shown that familiarity with morphemic patterns helps children to reasonably guess the meanings of unfamiliar words and is a powerful tool in vocabulary acquisition (see, e.g., Bertram et al., 2000). Morphology also provides reading strategies for correctly decoding and spelling unfamiliar words (Verhoeven and Perfetti, 2003, 2011). Knowledge of word morphology develops early in children, confirming that morphological structure is one of the main organizing principles of the mental lexicon. Morphological awareness improves with age and in each subsequent grade is more predictive of both reading and spelling achievement in children exposed to different orthographies (e.g., Mann and Singson, 2003; Berninger et al., 2010; Casalis et al., 2011). Morphological awareness usually predicts unique variance in addition to phonological awareness and has different degrees of association with word recognition (and spelling) in different scripts (McBride-Chang et al., 2003, 2005). Knowledge of derived words, in particular, may contribute to both reading and spelling achievement in older children, such as 6th graders (see, e.g., for Dutch, Rispens et al., 2008). Overall, access to morphemic constituents of words fosters reading and spelling performance in the course of literacy acquisition in several languages that vary for morphological richness and orthographic transparency (Verhoeven and Perfetti, 2011).

The effect of morphology on reading in Italian has received a good deal of attention in the last decade. A series of studies demonstrated that morpheme-based reading is available and efficient in Italian developing readers. In Italian, morphology has been found to have a main effect on reading fluency. Both typically developing Italian children ranging from 2nd to 7th grade and children with dyslexia read aloud pseudowords made up of a root and a derivational suffix (e.g., *donn-ista*, “woman-ist”) faster than pseudowords that did not include morphemes (e.g., *dennosto*, Burani et al., 2002, 2008; Traficante et al., 2011). Analogously, words with a morphological structure (e.g., *cass-iere*, “cashier”) were read faster than simple words (e.g., *cammello*, “camel”) of the same length and frequency. Morphological facilitation of word reading speed was found in the youngest readers (2nd graders) and in children with dyslexia, but it was limited to low-frequency words in older skilled children (Burani et al., 2008; Marcolini et al., 2011; see also, for a review, Burani, 2010). The facilitation induced by morphology on the reading performance of less skilled readers may reflect access to lexical reading units (morphemes) that are shorter than the whole word when this reading unit is too long and complex for the reader. Morphemes (specifically roots and suffixes) can be efficient reading units because they have an intermediate grain size between single letters—which entail extremely slow and analytical sublexical processing—and the word—which for beginning readers and children with dyslexia is usually too large a unit to be processed as a whole. By contrast, for skilled readers who master lexical reading of familiar word units, recourse to morphemic units is beneficial only for low frequency words. In this case, morphemes (roots and affixes) usually have a higher frequency than the word in which they occur. Therefore, access to morphemes may facilitate lexical reading for a low-frequency word that otherwise would probably not be represented as a whole in the mental lexicon.

Italian has a transparent orthography and knowledge of morphology is not necessary for assigning the correct pronunciation to print or for correct spelling. This is different from opaque orthographies, such as English, Danish and French, in which word spelling is to some degree morphologically governed and knowledge of morphemes may help to assign the correct pronunciation or to make the appropriate choice between graphemic alternatives for spelling a word (see, e.g., Seymour, 1997; Verhoeven and Perfetti, 2003; Pacton and Deacon, 2008 and see below for examples). As a consequence, in Italian the impact of morphological structure on reading accuracy is weaker than that on reading speed. However, the presence of morphemes in a stimulus positively affected reading accuracy in the case of novel (pseudo-) words (Burani et al., 2002, 2008; Traficante et al., 2011), but not in the case of words, irrespective of word frequency or reading skill (Burani et al., 2008; Marcolini et al., 2011).

As to word spelling, the effect of morphological structure in opaque orthographies has been studied in relation to the existence of two different spelling procedures, one based on phonology-to-orthography conversion rules (Patterson, 1986; Tainturier and Rapp, 2000) and the other relying on access to word-specific memories in the orthographic lexicon (e.g., Barry, 1994). Within this framework, morphosyntax has been considered a third source of information for spelling. In opaque orthographies, morphological information may contribute to spelling accuracy in several ways. For example, in English the use of morphological information allows: (1) choosing between several possible spellings of a given sound (e.g., the vowel /e/ in *health* can be spelled correctly if one knows the spelling of its root, *heal*); (2) spelling certain words for which phonology is misleading (the past tense of regular verbs ends in –*ed* although their pronunciation can be /d/, /t/ and /Id/); and (3) spelling cases that are morphologically distinct although phonologically identical, as in the case of the apostrophe (*boys*, *boy’s* and *boys’* sound the same but are spelled differently based on their morphological structure). Especially in the latter case, morphosyntactic information is necessary to to spell correctly because the use of markers cannot be retrieved from memory but depends on the syntactic context in which they occur. Evidence of the useful role of morphology in spelling mainly regards English and French. Many studies highlighted that children learning to spell in these two languages use various sources of morphosyntactic information to spell correctly (for a review see Pacton and Deacon, 2008). However, there is no agreement about the timing (early vs. late) of children’s use of morphological information.

In a longitudinal study, Nunes et al. (1997) examined the appreciation of morphological conventions such as those required for the correct spelling of regular past tense verbs in 6- to 9-year-old English children. The authors used a dictation task with regular past tense verbs (ending in –*ed* as in *called* and *dressed*), irregular past tense verbs (endings spelled phonetically as in *found* and *felt*), and non-verbs ending with /d/ and /t/ (such as in *bird* and *soft*). Four developmental stages were identified: in the first stage children wrote /d/ and /t/ endings phonetically, irrespective of stimulus type; then, they generalized the –*ed* ending to grammatically inappropriate words, as in spelling the adjective (e.g., *sofed* for *soft*). Later these generalizations applied to grammatically appropriate words (verbs) but incorrectly to irregular verbs (e.g., *keeped* for *kept*), and finally they were properly confined to regular past tense verbs. The authors concluded that children grasp the morphological principles of spelling only at late stages of literacy.

Other studies (e.g., Treiman et al., 1994; Kemp, 2006) found that very young children (5–8 years old) were able to use the principle of root consistency, although not to its full extent. Kemp (2006) examined whether young children used their knowledge of the spelling of base words to spell inflected and derived forms. The author examined how children spelled the /z/ sound in one- and two-morpheme words. In the case of two-morpheme words, the different alternatives to represent the word-medial /z/ sound (e.g., *S, Z, ZZ*) can be determined by knowledge of the base form spelling. The author found that 5- to 9-year-olds were more accurate in representing the medial /z/ sound of words derived from base forms (e.g., *noisy* from *noise*) with respect to one-morpheme control words (e.g., *busy*). These findings support the view that English-speaking children identify and represent links of meaning between words relatively early and that morphological information is a resource used also by relatively young learners. Similar conclusions come from studies in French-speaking children. Sénéchal (2000) found that children even in the first year of formal schooling spelled words that have morphologically related words better than words that do not. More recently, in a study in 8-year-old children Pacton et al. (2013) found that a facilitation due to morphological relatedness was present also when they learned to spell new words.

In transparent orthographies with consistent phoneme-to-grapheme correspondences, such as Spanish, Finnish and Italian, the mapping of phonology onto orthography is in principle sufficient to spell most words correctly. However, there is evidence that morphological knowledge may have a role in spelling also in transparent orthographies. One piece of evidence comes from the study of Defior et al. (2008) in Spanish first- to third-grade children, in which the recourse to morphological information in spelling was investigated by capitalizing on one of the few conditions of non-transparency in oral-to-written mapping. In Andalusia, the region where the experiment was conducted, the final /*s*/ of words is not pronounced. Since the final /s/ marks plurals and the second person singular of verbs, its presence in the children’s spellings was considered an index of their adequate use of morphosyntactic knowledge. The study included two morphological conditions (high- and low-frequency plural nouns and second person verbs), and a lexical control condition (high- and low-frequency singular nouns ending in /*s*/, e.g., *martes* “Tuesday”). Results showed that although Spanish spelling relies mainly on phonology, morphological information is also a spelling resource: with low-frequency words, children’s spelling accuracy on verbs and plural nouns (items with morphologically motivated /*s*/ endings) was greater than that on control words with a final /*s*/ (not morphologically motivated). However, the results also showed that the children did not use morphology systematically: in high-frequency words, they used fewer /*s*/ endings in plural nouns than in uninflected control words.

To our knowledge, no study on Italian has investigated the role of morphology in spelling. However, a recent study in first- to eighth-grade Italian typically developing readers (Notarnicola et al., 2012) reported several findings of interest in this respect: *(i*) in agreement with the hypothesis that reliance on the different procedures depends on the degree of regularity of an orthography (for reviews, see Sprenger-Charolles, 2003; Caravolas, 2004), in Italian main reliance on the sublexical phoneme-to-grapheme spelling procedure was found in all grades; (*ii*) data also showed very early reliance on the lexical procedure, with a lexicality effect (regular words spelled better than pseudowords) and an early positive influence of a lexical-semantic variable, such as word age-of-acquisition, on ambiguous word spelling found in first graders; and *(iii*) data generally supported the view of an interaction between lexical and sublexical spelling processes in Italian children. Results showed a pattern of correlations that was generally consistent with the view that spelling regular words benefits from the cooperation of both spelling procedures, with sublexical processing assisting accuracy in spelling lexical items.

In the cited study on Italian, no morphologically complex words were used. Consequently, the impact of morphology on spelling could not be estimated. However, it can be conjectured that, similarly to what happens in reading long unfamiliar stimuli, access to morphemes might help Italian children recognize lexical chunks of information and use them for (morpho-) lexical spelling, thus bypassing the use of single phoneme-to-grapheme correspondences. Thus, the spelling of long and complex words might benefit from the possibility of segmenting the phoneme-grapheme array into units, such as morphemes, that are meaningful and more coherent than single phonemes or syllables. Some support to the view that morphemes may provide an efficient principle for stimulus segmentation comes from an interesting study by Lehtonen and Bryant (2005) on Finnish, a richly inflected language with highly transparent orthography. The authors used two-morpheme words in which target clusters of letters (the sequences LL and SS) appeared in different morphemes of the words, either in the root (unbound morpheme) or in the inflection for case. In Finnish, case inflections are a more prominent part of morphology than derivation, because they occur in nouns, adjectives, pronouns and numerals. The authors tested children at two different times during the first year of school and found that by the end of the year they began to spell target clusters better in case inflections than in word roots, which suggested emerging sensitivity to the morphological structure of words in spelling. Similar results were found for pseudowords: letter clusters occurring in endings corresponding to case inflections were spelled with greater accuracy than those occurring in pseudo-roots, suggesting that case-like endings prompted morphological parsing during spelling. According to the authors, the facilitation arises because the children’s mental lexicon is organized in morphemes and case inflections are solidly acquired and represented in the mental lexicon due to the high frequency with which they occur. This in turn helps the subsequent parsing of words into their constituent morphemes, favoring the oral-to-written transcription process.

In the present study we investigated the effects of morphology on both reading and spelling accuracy of pseudowords and words in typically developing children in different grades, i.e., in third and fifth grade. For both reading and spelling, we expected that pseudowords made up of familiar morphemes (roots and derivational suffixes) would be read and spelled better than matched pseudowords that did not include any morphemic constituent. The expected findings would confirm those already obtained for reading (see Burani et al., 2002, 2008; Traficante et al., 2011) and would extend them to spelling. As to words, in preceding studies on Italian no evidence was found of an effect of morphological structure on reading accuracy. However, preceding studies on word reading either involved words of medium frequency (Burani et al., 2008), or, when low-frequency words were investigated, readers were in 6th–7th grade. Thus, we still do not know whether the presence of familiar morphemes in a low-frequency word favors reading (and spelling) accuracy in children as young as 3rd and 5th graders who might not yet possess a lexical representation for low-frequency words. In the present study, children’s reading and spelling performance on low-frequency morphologically complex words was compared to their performance on words with no derivational structure. In order to better qualify the effect of morphology, two types of morphologically complex words were investigated: words made-up of high-frequency morphemes (root and suffix) and words with low-frequency morphemes. Some studies have found that English-speaking children in 3rd to 6th grades read aloud derived words with a high frequency base more accurately than derived words with a low frequency base matched for surface frequency (Mann and Singson, 2003; Carlisle and Stone, 2005; Deacon et al., 2011). The new experimental contrasts adopted here, in which both types of morphologically complex words (i.e., including either high-frequency or low-frequency morphemes) were compared to words that did not include morphemes, allowed us to investigate an issue that has never been studied in Italian children. Higher accuracy was expected for morphological words including high-frequency morphemes as compared to words including low-frequency roots and suffixes and words not decomposable into morphemes. The advantage for words including high-frequency morphemes was expected to hold for both 3rd and 5th graders.

Administration of the same pseudowords and words for both reading and spelling allowed us to directly compare the children’s performance on both tasks. Overall, we expected that morphological knowledge would enhance not only reading but also spelling performance in Italian by facilitating the parsing process of the stimulus by retrieving lexical units smaller than the whole stimulus. Similarly to what has been observed for morpheme-based reading, morphological facilitation in spelling was expected to be evident for pseudowords, irrespective of the children’s reading ability. For low-frequency words, we expected that only those made up of high-frequency morphemes would result in a morphological benefit in spelling, with no substantial differences between children in different grades.

## Materials and method

### Participants

Participants were selected during screening activities, as part of a research agreement between the University of Bari and a local public primary school. The study was conducted according to the principles of the Helsinki Declaration and was approved by the school authority (Teaching body). Parents were informed of the screening activities and had to approve their child’s participation. All data concerning individual performances were analyzed strictly for research purposes.

Participants were typically developing readers and spellers selected according to the following criteria: *(i)* normal reading speed and accuracy on a standard reading test (MT reading test, Cornoldi and Colpo, 1998; see paragraph Reading Assessment), *(ii)* normal spelling performance on a standard spelling test (DDO Test for the Diagnosis of Developmental Dysgraphia, Angelelli et al., 2008; see paragraph Spelling Assessment ); and *(iii)* normal performance on a nonverbal general intelligence test (Raven’s Colored Progressive Matrices, CPM; i.e., above the 10th percentile for age range according to normative Italian data by Pruneti et al., 1996). Participants included 15 children in 3rd grade (7F, 8M; mean age = 8.65 year, sd = 0.27) and 15 children in 5th grade (7F, 8M; mean age = 10.34 year, sd = 0.38), matched one-to-one for gender and performance on Raven’s CPM intelligence test (*z* scores; *F*_(1,29)_ = 0.00, ns).

Data pertaining to the 3rd and 5th grade children’s performance on Raven’s CPM, the MT reading tasks (speed and errors) and the spelling test are summarized in Table 1. As reported in the Table, 5th grade children, compared to 3rd grade children, performed better in terms of reading speed and reduced error rates in reading as well as in all spelling subsets. Both groups of children performed close to normative data (*z* scores about zero) for Raven’s CPM (3rd grade children: *z* = −0.38; 5th grade children: *z* = −0.40), reading speed (3rd grade children: *z* = −0.22; 5th grade children: *z* = −0.43), reading accuracy (3rd grade children: *z* = 0.28; 5th grade children: *z* = −0.44) and for the total spelling task (3rd grade children: *z* = −0.32; 5th grade children: *z* = 0.07), indicating only marginal deviations from the same-age normative sample.

**Table 1:** **Mean (and SD) of 3rd and 5th grade children on the intelligence test (Raven’s Colored Progressive Matrices), the MT Reading test (Cornoldi and Colpo, 1998), and the writing task (Angelelli et al., 2008)**.

	3rd grade children		5th grade children	
	Mean	SD	Mean	SD
Raven’ CPM accuracy (*n* = 36)	24.20	3.76	28.47	4.14
MT reading speed	38.98	9.54	26.06	4.51
MT reading errors	6.30	2.66	3.20	1.62
Regular words 1:1 spelling accuracy (*n* = 70)	22.93	2.55	24.87	0.35
Context-sensitive words spelling accuracy (*n* = 10)	68.53	1.06	69.40	0.83
Ambiguous words spelling accuracy (*n* = 55)	9.60	0.83	9.87	0.35
Pseudowords spelling accuracy (*n* = 25)	45.40	4.21	50.40	3.38
Total spelling accuracy (*n* = 160)	146.47	6.40	154.53	3.76

*Note: Regular words 1:1 = words with one-sound-to-one-letter correspondence; context-sensitive words = words requiring the application of context-sensitive sound-to-spelling rules; ambiguous words = words with unpredictable transcription along the phonology-to-orthography conversion routine*.

### Reading assessment

Reading level was assessed using a standard reading achievement test (i.e., the MT Reading test, Cornoldi and Colpo, 1998). Participants read aloud a meaningful text passage within a 4-min time limit; speed (time in seconds per number of syllables read) and accuracy (number of errors, adjusted for the amount of text read) were computed. Stimulus materials and related reference norms varied depending on school grade. Raw scores were converted to *z* scores according to standard reference data. Normative data for third and fifth graders were based on 285 and 305 children, respectively (Cornoldi and Colpo, 1998).

### Spelling assessment

The participants’ spelling abilities were tested with a standard *spelling to dictation test* (DDO test, Angelelli et al., 2008), which consisted of four sections:

Section A: regular words with full one-sound-to-one-letter correspondence (*N* = 70).

Section B: regular words requiring the application of context-sensitive sound-to-spelling rules (*N* = 10). In Italian, context-sensitive rules are required when the orthographic transcription of a phoneme depends on the following letter. For example, the phoneme /k/ is spelled C, when followed by a consonant (e.g., in *clima* (/klima/ “climate”) or by A, O, U (e.g., in *casa /*kaza/ “home”; *cono* /kono/ “cone”; and *cubo* /kubo/ “cube”) and CH when followed by E or I (e.g., in *chilo*/kilo/ “kilogram”).

Section C: ambiguous words (words with two or more possible transcriptions along the phonology-to-orthography conversion routine; (e.g., words containing the syllables /tʃe/, /ʃe/ and */*dʒe/, which may or may not require an I (e.g., /ʃentsa/ “science” is spelled *scienza* and not *scenza*, while /ʃena/ “scene” is spelled *scena* and not *sciena*) (*N* = 55).

Section D: pseudowords with one-sound-to-one-letter correspondence (*N* = 25).

Words with one-sound-to-one-letter correspondence and pseudowords were controlled for orthographic complexity (i.e., number of consonant clusters, double consonants) and length.

Normative data are available for first- to eighth-grade children (Angelelli et al., 2008). Reference data for third and fifth graders are based on 95 and 105 children, respectively. Raw scores were converted to *z* scores.

### Experimental lists

Different sets of low-frequency words and pseudowords were created.

Words: Three sets of 15 low-frequency words (Istituto di Linguistica Computazionale, CNR, unpublished) were used. Words in the first set (e.g., bruttezza, “ugliness”) consisted of a root (brutt- “ugly”) and a derivational suffix (-ezza, “ness”), which were both of high frequency (HD). Words in the second set (*e.g*., agrumeto, “citrus grove”) consisted of a root (agrum-, “citrus plant”) and a derivational suffix (-eto, indicating a place where trees or flowers grow), which were both of low frequency (LD). The third set of words included simple non-derived words (ND) (e.g., aragosta, “lobster”). The three sets of words were matched for word frequency and did not differ for relevant psycholinguistic variables such as length (number of letters), consonant clusters, geminate letters, number of contextual rules and bigram frequency (all *p*s > 0.05). As expected, the first and the second set were different for root frequency (*F*_(1,28)_ = 17.73, *p* < 0.001) and suffix frequency (*F*_(1,28)_ = 15.79, *p* < 0.001). All words (with frequency values) are reported in Appendix A.

Pseudowords: Two sets of 16 pseudowords of three to four syllables (length range: 8–10 letters) were generated: pseudowords in the first set were morphologically complex (root + suffix) and consisted of a root and a derivational suffix (R^+^S^+^) in a combination that does not exist in the Italian language (e.g., lampadista, constituted by the bound root lampad-, meaning “lamp” and the suffix –ista, “-ist”). Pseudowords in the second set (non-root + non-suffix) were made up of orthographic sequences that did not correspond to any existing Italian root or suffix (R^−^S^−^) (*e.g*., livonosto). Analogously to the ND words, the pseudowords in the latter set had no morphological structure. The two sets of morphemic and non-morphemic pseudowords were matched for number of contextual rules, consonant clusters, geminate letters, length (in letters) and bigram frequency (all *F*s < 1). The two sets of pseudowords were also matched for the frequency of the final orthographic sequence, which corresponded either to a real suffix in the R^+^S^+^ set or a non-suffix in the R^−^S^−^ set. All pseudowords with frequency values of constituent parts are reported in Appendix B.

We added 43 filler stimuli to the list, that is, 15 non-morphologically complex words and 18 pseudowords; half were morphologically complex and half were simple. A total of 110 stimuli were presented to each child for dictation; half were words and half were pseudowords, half were morphologically complex and half were simple. This list of words and pseudowords was intended to favor lexical reading without explicitly inducing morphological decomposition.

### Procedure

For the reading condition words and pseudowords were randomized and presented in three blocks of either 36 or 37 items each, using different random orders. Stimuli were displayed at the center of the computer screen; they were printed in black lower case (Arial font, 24 pt). Each trial consisted of the following sequence: a fixation point for 500 ms; a blank stimulus for 250 ms; the stimulus, which remained visible until the onset of pronunciation. Participants read each stimulus aloud as accurately as possible. Mispronunciation errors were recorded and noted by two experimenters, who verified their annotations at the end of the experimental sections. The experimental sections were preceded by a training block of 10 stimuli, that is, five words and five pseudowords.

For the spelling condition, words and pseudowords were randomized and administered in a spelling-to-dictation task. The examiner read each item aloud in a neutral tone without emphasizing the presence of possible orthographic difficulties. To ensure that the children had correctly perceived the items, the examiner asked them to repeat each one before they wrote it down in capital letters. No feedback was provided on the correctness of the written response. Pauses were allowed if requested. Spontaneous corrections were accepted.

The reading and spelling tests were administered with an interval of about 20 days between them (were administered about 20 days apart). The order of the tasks was balanced in the experimental sample: half of the children performed the reading task first and then the spelling test, and the other half performed the tasks in reverse order; children were randomly assigned to the first or second sub-group. They were tested individually in a quiet room at their school.

### Data analysis

Reading and spelling accuracy were analyzed with Logistic Mixed Effect Models (Guo and Zhao, 2000; Quené and van den Bergh, 2008) by means of SPSS 22.0 statistics software. Logistic Mixed Effect Models were used to control for the presence of a floor effect as well as for item and participant variability. In this analysis the dependent variable was accuracy on each item of each participant in each experimental condition/sample; thus, the number of observations was very high.

Data on words and pseudowords were analyzed separately. In both analyses, *Task* (reading vs. spelling), *Grade* (3rd vs. 5th grade) and *Morphology* were entered as fixed factors, and *Items* and *Participants* were entered as Random factors. Note that in the case of words the effect of *Morphology* refers to words made up of high-frequency roots and high-frequency derivational suffixes [HD], low-frequency roots and low-frequency derivational suffixes [LD] and non-derived words [ND]; in the case of pseudowords, *Morphology* refers to pseudowords made up of real roots and derivational suffixes [R^+^S^+^] and pseudowords, including orthographic sequences that did not correspond to any existing Italian root or suffix [R^−^S^−^]. Interactions were explored by means of pairwise *post-hoc* tests.

Although comparisons between word sets for word frequency were non-significant (see paragraph Experimental Lists), words in the LL condition showed some rather unbalanced word frequencies relative to the other two sets. Therefore, to ensure that the results obtained were not a by-product of some word frequency differences between sets, a second analysis was performed in which word frequency was entered as a covariate.

### Results

#### Words

The Logistic Mixed Effect Model showed a significant effect of *Task* (*F*_(1,2732)_ = 20.64, *p* < 0.0001), *Grade* (*F*_(1,2732)_ = 11.80, *p* < 0.001) and *Morphology* (*F*_(1,2732)_ = 4.47, *p* < 0.01), with a higher error rate in reading with respect to spelling (9.3% vs. 4.3%, respectively), in 3rd compared to 5th grade children (9.5% vs. 4.2%), and in ND and LD words with respect to HD words (8.8% and 8.5% vs. 3.3%, respectively). The *Morphology × Task* interaction (*F*_(1,2732)_ = 10.76, *p* < 0.0001) was significant, showing an effect of morphology in reading (*p* < 0.0001) but not in spelling. Exploration of means revealed that HD words were read significantly better than LD and ND words (*p* < 0.001 and *p* < 0.01, respectively) and that LD words were read worse than ND words (*p* < 0.05). Furthermore, *post-hoc* analysis showed that the HD condition led to comparable error percentages in reading and spelling, but the LD and ND words had a significantly higher error rate in reading than in spelling (for LD, difference between reading and spelling = 17.5%, *p* < 0.0001; for ND, difference between reading and spelling = 4.2%, *p* < 0.05).

**Figure 1:**
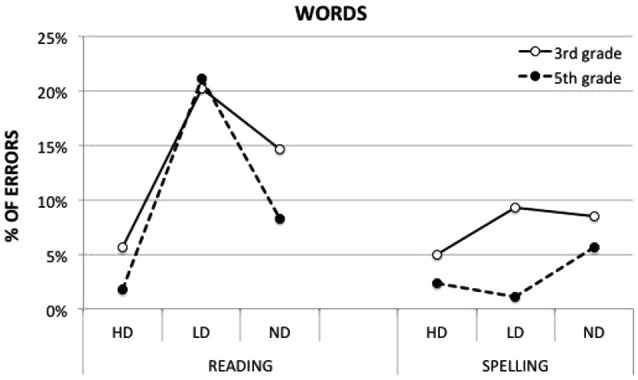
**Reading and spelling performance (percentage of errors) on words with high-frequency roots and suffixes (HD), low-frequency roots and suffixes (LD) and non-derived words by typically developing 3rd and 5th grade children**.

The *Task × Morphology × Grade* interaction (*F*_(1,2732)_ = 5.57, *p* < 0.01) was significant. Figure 1 shows how morphology modulates reading and spelling performance for words in 3rd and 5th grade children. Table 2 reports mean error percentages (and standard errors values) as a function of task and stimulus type. The effect of morphology was significant in reading for both 3rd and 5th graders (*p* < 0.01 and *p* < 0.0001, respectively), but not in spelling (either for 3rd or 5th graders). Exploration of means showed that in reading both 3rd and 5th grade children performed more incorrectly on LD than HD words (difference = 14.6%, and 19.3% in 3rd and 5th grade, respectively, at least *p* < 0.01); and on ND compared to HD words (difference = 9.0% and 6.5%, in 3rd and 5th grade, respectively, at least *p* < 0.05); only 5th graders showed a difference also between LD and ND (difference = 12.8%, *p* < 0.05), indicating that the LD condition was the most difficult one. In spelling, both groups had very low and comparable percentages of errors on HD and LD words; the only significant effect was in 5th graders, who spelled ND words less correctly than morphologically complex stimuli (ND vs. LD diff. = 4.5%, *p* < 0.05). Finally, progressing from 3rd to 5th grade, errors decreased for HD words (*p* < 0.05) and ND words (*p* = 0.06) in reading and for LD words (*p* < 0.01) in spelling. A comparison between reading and spelling performances showed significantly lower accuracy in reading than in spelling only for the LD condition in both 3rd and 5th graders (*p* < 0.01 and *p* < 0.0001, respectively).

**Table 2:** **Mean percentage of errors (and SE) of 3rd and 5th grade children in reading and spelling experimental words**.

	Reading			Spelling		
	HD	LD	ND	HD	LD	ND
3rd grade	5.6 (2.0)	20.2 (4.9)	14.6 (4.0)	5 (1.8)	9.3 (2.8)	8.5 (2.7)
5th grade	1.8 (0.9)	21.1 (5.0)	8.3 (2.6)	2.3 (1.1)	1.1 (0.7)	5.6 (2.0)

*Note: HD = words with high frequency roots and suffixes; LD = words with low frequency roots and suffixes; ND = non-derived words*.

The random effects of *Items* (*Z* = 3.52; *p* < 0.0001) and *Participants* (*Z* = 2.15; *p* < 0.05) were significant.

When word frequency was added as a covariate in the analysis it approached significance (*F*_(1,2.732)_ = 3.45, *p* = 0.06). However, the effects of *Task (F*_(1,2732)_ = 20.64, *p* < 0.0001), *Grade* (*F*_(1,2732)_ = 11.80, *p* < 0.001), *Morphology* (*F*_(1,2732)_ = 4.47, *p* < 0.05), as well as the second level interaction *Task × Morphology × Grade* (*F*_(1,2732)_ = 5.57, *p* < 0.01) remained unchanged.

#### Pseudowords

**Figure 2:**
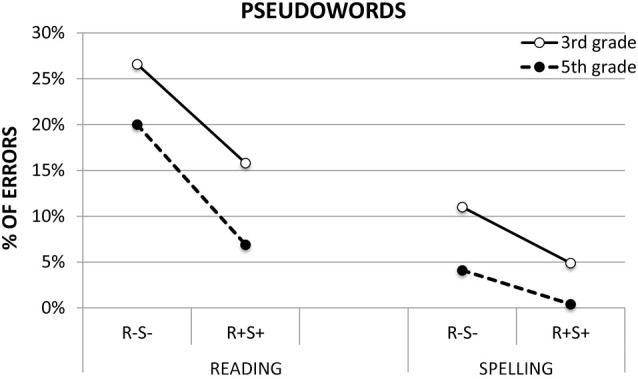
**Reading and spelling performance (percentage of errors) on pseudowords made up of real roots and derivational suffixes [R^+^S^+^] and pseudowords with orthographic sequences that do not correspond to any existing Italian root or suffix [R**^−^**S**^−^**] by typically developing 3rd and 5th grade children**.

Figure 2 shows the effect of morphology on the reading and spelling accuracy performance of 3rd and 5th grade children on pseudowords. Table 3 reports mean error percentages (and standard error values) as a function of task and stimulus type. The analyses indicated significant effects of *Task* (*F*_(1,1944)_ = 35.71, *p* < 0.0001), *Grade* (*F*_(1,1944)_ = 14.94, *p* < 0.0001) and *Morphology* (*F*_(1,1944)_ = 16.66, *p* < 0.0001). Exploration of the main effects showed higher error rates in reading (15.9%) than in spelling (3.1%) in 3rd graders compared to 5th graders (12.6% vs. 4.6%, respectively), and in R^−^S^−^ with respect to R^+^S^+^ pseudowords (12.9% vs. 4.0%, respectively).

**Table 3:** **Mean percentage of errors (and SE) of 3rd and 5th grade children in reading and spelling experimental pseudowords**.

	Reading		Spelling	
	R^−^S^−^	R^+^S^+^	R^−^S^−^	R^+^S
3rd grade	26.6 (3.6)	15.8 (2.8)	11 (2.3)	4.9 (1.5)
5th grade	20 (3.1)	6.9 (1.7)	4.1 (1.4)	0.4 (0.4)

*Note: R^−^S^−^ = pseudowords made up of existing roots and suffixes; R^+^S^+^ = pseudowords with any existing roots and suffixes*.

The *Grade × Task* interaction was significant (*F*_(1,1944)_ = 3.71, *p* < 0.05), showing higher percentages of errors in reading compared to spelling in both grades (3rd grade difference = 13.4%; 5th grade difference = 10.7%, at least *p* < 0.0001), with a larger difference between reading and spelling in 3rd grade children. Moreover, 3rd graders were less correct than 5th graders in both spelling (difference = 6.0%, *p* < 0.01) and reading (difference = 8.7%, *p* < 0.0001). The *Morphology × Grade* interaction was marginally significant (*F*_(1,1944)_ = 2.98, *p* = 0.08), indicating a significant effect of morphology in both groups, which was larger for 3rd graders whose error rates decreased from 17.4% to 8.9% passing from the R^−^S^−^ to the R^+^S^+^ conditions (difference = 8.5%, *p* < 0.0001); 5th graders’ errors decreased from 9.4% to 1.7% for R^−^S^−^ and R^+^S^+^, respectively (difference = 7.7%, *p* < 0.0001).

The random effects of *Items* and *Participants* were not significant (*Z*s about 1).

## Discussion

In the present study, we aimed to investigate whether there is evidence of an early use of morphological information in both reading and spelling in languages with transparent orthography, such as Italian, and whether the frequency of morphemes modulates the use of morphology in both tasks, an issue not yet investigated in Italian children. Results confirmed that morphological information is a useful resource in children’s reading and partially extended the evidence for morphological facilitation to the spelling process. For both tasks, they also indicated the conditions in which this facilitation occurs.

Morphology was helpful for both 3rd and 5th graders when they read and spelled pseudowords. Both younger and older children benefitted from the presence of morphological constituents when processing newly encountered stimuli; indeed, pseudowords made up of existing morphemes were read and spelled more accurately than non-morphemic pseudowords, irrespective of school level (with a somewhat higher advantage for younger than for older children). These results can be interpreted as a genuine morphological effect, rather than a generic “wordlikeness” effect, because we carefully controlled for familiarity of the chunks constituting the pseudowords. For instance, suffixes could not be considered more familiar chunks than orthographic sequences in the non-morphological set, because for the latter set of stimuli we selected final orthographic sequences that were as frequent as suffixes in the root + suffix pseudoword set. Furthermore, pseudowords in the two sets (morphological and non-morphological) were matched exactly for mean bigram frequency.

As to low-frequency words, morphological facilitation was present in reading but not in spelling. Words made up of high-frequency roots and suffixes were read better than non-derived ones by both 3rd- and 5th-grade children. However, a difference between groups emerged in reading words composed of low-frequency morphemes: while third graders read words with low-frequency morphemes at a comparable level of accuracy as non-derived words, 5th graders read words composed of low-frequency morphemes even worse than non-derived words. These data indicate that younger children do not rely on morphological parsing when morphemes are of low frequency, because these morphemes are unknown to them. Consequently, younger children treat words with low-frequency morphemes similarly to words that include no morphemes. By contrast, 5th graders may attempt morphological parsing also when reading words composed of low-frequency morphemes, but this attempt may actually result in more errors than in the case of non-derived words (which cannot be decomposed into morphemes). It can be speculated that in reading a word made up of low-frequency morphemes, the oldest children might occasionally succeed in accessing the root; however, after accessing the root the children may expect the higher frequency suffix which is present in the base word rather than the lower-frequency suffix actually present in the derived word. As a consequence, the combination of morphemes might lead to uncertainty or to the erroneous production of the base word. Thus, the worse performance of 5th graders on words with low-frequency morphemes than on all other words confirms the tendency of older children to rely on morpho-lexical reading.

The present data are consistent with the literature showing that morphology plays a role in reading in transparent orthographies, where in principle the regularity of the phoneme-to-grapheme mapping is sufficient to correctly process most words. Pseudoword data replicate those that emerged in several studies on Italian children. Burani et al. (2002) found an advantage in reading pseudowords composed of morphemes (root + suffix) compared to pseudowords without morphological structure in 3rd and 5th grade typically developing readers. Similar results were reported for 2nd grade and 6th–7th grade typically developing readers (Burani et al., 2008). Regarding words, in a first study Burani et al. (2008) used medium-frequency morphologically complex words and found that only children with dyslexia and younger typically developing children (2nd graders) benefitted from the presence of morphemes in reading words relative to simple words. By contrast, 6th–7th graders and adult skilled readers showed no difference in reading morphologically complex words vs. simple words (Burani et al., 2008). However, in a second study, Marcolini et al. (2011) showed that word frequency can modulate morpheme-based reading in skilled readers (6th–7th graders), facilitating the reading of low- but not high-frequency morphologically complex words. According to the authors, when a unit larger than the morpheme (i.e., the whole word) is available because it has a high frequency, morphemic parsing does not necessarily facilitate processing. Parsing a word into morphemes entails both benefits and costs, and costs may prevail over benefits when there is the alternative possibility of reading the word as a whole (Schreuder and Baayen, 1995; Traficante and Burani, 2003). Consequently, for skilled readers morphemic parsing may be an efficient strategy only with new or unfamiliar words, for which no whole-word representation is available. The present reading data are consistent with this interpretation: all words used in our study were of low frequency and we observed morphological facilitation. Moreover, a negative effect of morphological parsing in skilled readers also emerged, with worse performance on morphemic words made up of low-frequency roots and suffixes than on simple non-derived words.

A new finding of this study was the presence of morphological facilitation in the spelling of stimuli with regular transcription; however, the facilitation was limited to pseudowords. In fact, even if morphological parsing was attempted in spelling words, it did not produce appreciable effects; however, some facilitation was present in 5th grade children for morphemic words. We interpreted these findings as follows: The facilitation for novel stimuli may arise from parsing and subsequent access to smaller (than the whole stimulus) and more manageable lexical units. For developing readers, in fact, exposure to these frequently occurring chunks of sound and meaning in speech and their corresponding orthographic patterns in writing could allow morphemes to become relatively independent spelling units. This would enable children to process them correctly avoiding time-consuming and error-prone phoneme-grapheme analysis. The different results obtained for pseudoword and word spelling—with morphological facilitation present only in the former—give some indications. In spelling a word, morphological parsing may be less influential than in pseudoword spelling because the whole-word spelling procedure—together with the sublexical phoneme-to-grapheme conversion routine (and their mutual interaction)—may have a relevant role. This could explain the absence of a significant modulation of morphology on word spelling. Consistent with this hypothesis is the finding of very early signs that is, from the first years of schooling, of lexical spelling (Notarnicola et al., 2012), with first graders already able to spell correctly 60% of the words that require reliance on lexical orthographic representations. Overall, the morphological facilitation found in spelling, although prevalent for non lexical stimuli, is consistent with the conclusions reached by the few studies conducted in transparent orthographies (Lehtonen and Bryant, 2005; Defior et al., 2008). In those studies, morphological knowledge was found to be exploited in different experimental conditions by children learning to spell.

A final result of our study deserves some comment. In both 3rd and 5th grade children we found higher accuracy in spelling than in reading when the same sets of stimuli were compared. We believe that this difference in error rates may be due to task-specific processes. It is worth noting that, unlike reading, in spelling under dictation there is enough time to activate a word representation in the mental lexicon because the word is fully available to the speller before starting the process of writing it. Thus, an additional locus of facilitation is the activation of the spoken lexical form (see, e.g., Chua and Liow, 2014). In addition, the spelling response is produced without time pressure. In other words, in spelling the decoding phase is separate from the transcoding phase and usually neither process is under time pressure. Therefore, especially with regular stimuli, in lexical and sublexical processes (which may produce converging information) there is enough time for successful integration in spelling, thus leading to high accuracy. Conversely, reading is an online task in which the time lapse between stimulus recognition and response is very short (thus the stimulus decoding has to be done rapidly) and online corrections become reading errors. This could explain the lower number of errors in spelling with respect to reading, especially in those conditions in which the morphemic strategy is riskier, such as the case of words with low-frequency constituents. In the latter condition, that is, in the only condition that showed a significant difference between reading and spelling, online corrections led to errors in reading but not in spelling, where the response could be delayed with respect to the decoding phase and online corrections could be successfully incorporated.

The data that emerged from the present study have clear empirical implications. The facilitatory effect of morphology in reading and in spelling new words could be used to enrich standard teaching methods and rehabilitation strategies in the case of learning disabilities. Regarding reading, in our study only accuracy was considered. However, previous reports showed that morphology enhanced reading fluency (see Burani, 2010, for a review) in Italian children with dyslexia who are characterized by a prevalent deficit of reading speed (Zoccolotti et al., 1999). In the present study we found small but reliable effects in the spelling of regular stimuli of 3rd and 5th grade children. Studies in larger populations are needed to confirm the present data. However, considering that the transcription of regular stimuli is optimized very early in Italian (see Notarnicola et al., 2012), larger facilitatory effects might be found in younger learners. Furthermore, considering that some errors on ambiguous words are still present in 8th grade typically developing children and that a selective impairment of ambiguous word transcription characterizes the writing deficit of Italian children with learning disabilities (Angelelli et al., 2004, 2010), we believe that recourse to morphology is particularly helpful in situations of spelling ambiguity (e.g., knowledge of the spelling of SCIENZA “science” may facilitate the spelling of SCIENZIATO “scientist”, FANTASCIENZA “science fiction”, etc.). In this sense the introduction of morphemes in teaching materials and an emphasis on morphemic strategies could be particularly useful in the early phases of literacy acquisition as well as in children with learning disabilities (see, e.g., Elbro and Arnbak, 1996; Traficante, 2012). Explicit training using morphological strategies might induce children to identify patterns of letters that are consistent among several words and foster the processing of units that are larger than single phonemes/graphemes. However, future research is needed to further explore the possible benefits of morphological training, especially in transparent orthographies.

Overall the present study extends the role of morphology from reading to the spelling of newly encountered stimuli in a language with transparent orthography (Italian) and highlights the possible role of morphological knowledge in promoting literacy acquisition.

## Conflict of interest statement

The authors declare that the research was conducted in the absence of any commercial or financial relationships that could be construed as a potential conflict of interest.

## References

[B1] Angelelli P. Judica A. Spinelli D. Zoccolotti P. Luzzatti C. (2004). Characteristic of writing disorders in Italian dyslexic children. Cogn. Behav. Neurol. 17, 18–31. 10.1097/00146965-200403000-00003 15209222

[B2] Angelelli P. Notarnicola A. Costabile D. Marinelli C. V. Judica A. Zoccolotti P. (2008). DDO- Diagnosi dei Disturbi Ortografici in Età Evolutiva [Diagnosis of Orthographic Deficits in Childhood]. Trento: Erickson.

[B3] Angelelli P. Notarnicola A. Judica A. Zoccolotti P. Luzzatti C. (2010). Spelling impairment in Italian dyslexic children: phenomenological changes in primary school. Cortex 46, 1299–1311. 10.1016/j.cortex.2010.06.015 20688322

[B4] Barry C. (1994). “Spelling routes (or roots or rutes),” in Handbook of Spelling: Theory, Process and Intervention, eds Brown G. D. A. Ellis N. C. (Chichester: John Wiley), 27–49.

[B5] Berninger V. W. Abbott R. D. Nagy W. Carlisle J. (2010). Growth in phonological, orthographic and morphological awareness in grades 1 to 6. J. Psycholinguist Res. 39, 141–163. 10.1007/s10936-009-9130-6 19826956

[B6] Bertram R. Laine M. Virkkala M. M. (2000). The role of derivational morphology in vocabulary acquisition: get by with a little help from my morpheme friends. Scand. J. Psychol. 41, 287–296. 10.1111/1467-9450.00201 11131950

[B7] Burani C. (2010). Word morphology enhances reading fluency in children with developmental dyslexia. Lang. Lang. 9, 177–198 10.1418/33326

[B8] Burani C. Marcolini S. De Luca M. Zoccolotti P. (2008). Morpheme-based reading aloud: evidence from dyslexic and skilled Italian readers. Cognition 108, 243–262. 10.1016/j.cognition.2007.12.010 18262178

[B9] Burani C. Marcolini S. Stella G. (2002). How early does morpho-lexical reading develop in readers of a shallow orthography? Brain Lang. 81, 568–586. 10.1006/brln.2001.2548 12081423

[B10] Caravolas M. (2004). Spelling development in alphabetic writing systems: a cross-linguistic perspective. Eur. Psychol. 9, 3–14 10.1027/1016-9040.9.1.3

[B11] Carlisle J. F. Stone C. A. (2005). Exploring the role of morphemes in word reading. Read. Res. Q. 40, 428–449 10.1598/rrq.40.4.3

[B12] Casalis S. Deacon S. H. Pacton S. (2011). How specific is the connection between morphological awareness and spelling? A study of French children. Appl. Psycholinguist. 32, 499–511 10.1017/s014271641100018x

[B13] Chua S. M. Liow S. J. R. (2014). The locus of word frequency effects in skilled spelling-to-dictation. Q. J. Exp. Psychol. (Hove) 67, 1720–1741. 10.1080/17470218.2013.868915 24303871

[B14] Cornoldi C. Colpo G. (1998). Prove di Lettura MT. Guida all’uso [The MT Reading Test: User Manual]. Firenze, IT: Organizzazioni Speciali.

[B15] Deacon S. H. Whalen R. Kirby J. R. (2011). Do children see the danger in dangerous? Grade 4, 6 and 8 children’s reading of morphologically complex words. Appl. Psycholinguist. 32, 467–481 10.1017/s0142716411000166

[B16] Defior S. Alegría J. Titos R. Martos F. (2008). Using morphology when spelling in a shallow orthographic system: the case of Spanish. Cogn. Dev. 23, 204–215 10.1016/j.cogdev.2007.01.003

[B17] Elbro C. Arnbak E. (1996). The role of morpheme recognition and morphological awareness in dyslexia. Ann. Dyslexia 46, 209–240. 10.1007/bf02648177 24234273

[B18] Guo G. Zhao H. X. (2000). Multilevel modeling for binary data. Annu. Rev. Sociol. 26, 441–462 10.1146/annurev.soc.26.1.441

[B20] Kemp N. (2006). Children’s spelling of base, inflected and derived words: links with morphological awareness. Read. Writ. 19, 737–765 10.1007/s11145-006-9001-6

[B21] Lehtonen A. Bryant P. (2005). Active players or just passive bystanders? The role of morphemes in spelling development in a transparent orthography. Appl. Psycholinguist. 26, 137–155 10.1017/s0142716405050113

[B22] Mann V. Singson M. (2003). “Linking morphological knowledge to English decoding ability: large effects of little suffixes,” in Reading Complex Words: Cross-Language Studies, eds Assink E. Sandra D. (New York: Kluwer Academic/Plenum Publishers), 1–25.

[B23] Marcolini S. Traficante D. Zoccolotti P. Burani C. (2011). Word frequency modulates morpheme-based reading in poor and skilled Italian readers. Appl. Psycholinguist. 32, 513–532 10.1017/s0142716411000191

[B24] Marinelli C. V. Angelelli P. Notarnicola A. Luzzatti C. (2009). Do Italian dyslexic children use the lexical reading route efficiently? An orthographic judgment task. Read. Writ. 22, 333–351 10.1007/s11145-008-9118-x

[B25] McBride-Chang C. Cho J.-R. Liu H. Wagner R. K. Shu H. Zhou A.. (2005). Changing models across cultures: associations of phonological awareness and morphological structure awareness with vocabulary and word recognition in second graders from Beijing, Hong Kong, Korea and the United States. J. Exp. Child Psychol. 92, 140–160. 10.1016/j.jecp.2005.03.009 15904930

[B26] McBride-Chang C. Shu H. Zhou A. Wat C. Wagner R. K. (2003). Morphological awareness uniquely predicts young children’s Chinese character recognition. J. Educ. Psychol. 95, 743–751 10.1037/0022-0663.95.4.743

[B27] Nagy W. E. Anderson R. C. (1984). How many words are there in printed school English? Read. Res. Q. 19, 304–330 10.2307/747823

[B28] Notarnicola A. Angelelli P. Judica A. Zoccolotti P. (2012). The development of spelling skills in a shallow orthography: the case of the Italian language. Read. Writ. 25, 1171–1194 10.1007/s11145-011-9312-0

[B29] Nunes T. Bryant P. Bindman M. (1997). Morphological spelling strategies: development stages and processes. Dev. Psychol. 33, 637–649. 10.1037//0012-1649.33.4.637 9232379

[B30] Pacton S. Deacon S. H. (2008). The timing and mechanisms of children’s use of morphological information in spelling: a review of evidence from English and French. Cogn. Dev. 23, 339–359 10.1016/j.cogdev.2007.09.004

[B31] Pacton S. Foulin J. N. Casalis S. Treiman R. (2013). Children benefit from morphological relatedness when they learn to spell new words. Front. Psychol. 4:696. 10.3389/fpsyg.2013.00696 24109464 PMC3790073

[B32] Patterson K. E. (1986). Lexical but nonsemantic spelling. Cogn. Neuropsychol. 3, 341–367 10.1080/02643298608253363

[B33] Pruneti C. A. Fenu A. Freschi G. Rota S. Cocci D. Marchionni M. (1996). Aggiornamento alla standardizzazione italiana del test delle Matrici Progressive Colorate di Raven (CPM). [Update of the Italian standardization of Raven’s coloured progressive matrices]. Boll. Psicol. Appl. 217, 51–57.

[B34] Quené H. van den Bergh H. (2008). Examples of mixed-effects modeling with crossed random effects and with binomial data. J. Mem. Lang. 59, 413–425 10.1016/j.jml.2008.02.002

[B35] Rispens J. E. McBride-Chang C. Reitsma P. (2008). Morphological awareness and early and advanced word recognition and spelling in Dutch. Read. Writ. 21, 587–607 10.1007/s11145-007-9077-7

[B36] Schreuder R. Baayen R. H. (1995). “Modeling morphological processing,” in Morphological Aspects of Language Processing, eds Feldman L. B. (Hillsdale, N.J.: Erlbaum), 131–154.

[B37] Sénéchal M. (2000). Morphological effects in children’s spelling of French words. Can. J. Exp. Psychol. 54, 76–86. 10.1037/h0087331 10881392

[B38] Seymour P. H. K. (1997). “Foundations of orthographic development,” in Learning to Spell, eds Perfetti C. Rieben L. Fayol M. (Hillsdale, N.J.: Erlbaum), 319–337.

[B39] Sprenger-Charolles L. (2003). “Reading acquisition: cross linguistic data,” in Handbook of Children’s Literacy, eds Nunes T. Bryant P. (Dordrecht: Kluwer Academic Publishers), 43–65.

[B40] Tainturier M. J. Rapp B. (2000). “The spelling process,” in What Deficits Reveal about the Human Mind: A Handbook of Cognitive Neuropsychology, ed Rapp B. (Philadelphia, PA: Psychology Press), 263–289.

[B41] Thornton A. M. Iacobini C. Burani C. (1997). BDVBD Una Base di Dati Sul Vocabolario di Base Della Lingua Italiana [BDVDB: A Database for the Italian Basic Dictionary]. Roma: Bulzoni.

[B42] Traficante D. (2012). From graphemes to morphemes: an alternative way to improve skills in children with dyslexia. J. Res. Speech 2, 163–185.

[B43] Traficante D. Burani C. (2003). “Visual processing of Italian verbs and adjectives: the role of inflectional family size,” in Morphological Structure in Language Processing, eds Baayen H. R. Schreuder R. (Berlin: Mouton de Gruyter), 45–64.

[B44] Traficante D. Marcolini S. Luci A. Zoccolotti P. Burani C. (2011). How do roots and suffixes influence reading of pseudowords: a study of Italian children with and without dyslexia. Lang. Cogn. Process. 26, 777–793 10.1080/01690965.2010.496553

[B45] Treiman R. Cassar M. Zukowski A. (1994). What type of linguistic information do children use in spelling? The case of flaps. Child Dev. 65, 1318–1337. 10.2307/1131501 7982352

[B46] Verhoeven L. Perfetti C. (2003). Introduction to this special issue: the role of morphology in learning to read. Sci. Stud. Read. 7, 209–217 10.1207/s1532799xssr0703_1

[B47] Verhoeven L. Perfetti C. A. (2011). Morphological processing in reading acquisition: a cross-linguistic perspective. Appl. Psycholinguist. 32, 457–466 10.1017/s0142716411000154

[B48] Zoccolotti P. De Luca M. Di Pace E. Judica A. Orlandi M. Spinelli D. (1999). Markers of developmental surface dyslexia in a language (Italian) with high grapheme-phoneme correspondence. Appl. Psycholinguist. 20, 191–216 10.1017/s0142716499002027

